# Transgenerational Effects and Heritability of Folate Receptor Alpha Autoantibodies in Autism Spectrum Disorder

**DOI:** 10.3390/ijms26178293

**Published:** 2025-08-26

**Authors:** Richard E. Frye, Ira L. Cohen, Jeffrey M. Sequeira, Zoe Hill, Alina Espinoza, W. Ted Brown, Clifford Mevs, Elaine Marchi, Michael Flory, Edmund C. Jenkins, Milen T. Velinov, Edward V. Quadros

**Affiliations:** 1 Autism Discovery and Treatment Foundation, Phoenix, AZ 85050, USA; zoe@autismdiscovery.org (Z.H.); alina@autismdiscovery.org (A.E.); 2 Department of Psychology, New York State Institute for Basic Research in Developmental Disabilities, Staten Island, NY 10314, USA; ilcphd@gmail.com; 3 Department of Medicine, SUNY Downstate Medical Center, Brooklyn, NY 11203, USAedward.quadros@downstate.edu (E.V.Q.); 4 Department of Human Genetics, New York State Institute for Basic Research in Developmental Disabilities, Staten Island, NY 10314, USA; ted.brown@opwdd.ny.gov (W.T.B.); elaine.marchi@opwdd.ny.gov (E.M.); edmund.jenkins@opwdd.ny.gov (E.C.J.); 5 Pediatric Health Care, Staten Island, NY 10311, USA; 6 Research Design and Analysis Core, New York State Institute for Basic Research in Developmental Disabilities, Staten Island, NY 10314, USA; 7 Department of Genetics, Rutgers University, Piscataway, NJ 08854, USA; mv662@rwjms.rutgers.edu

**Keywords:** anticipation, autism spectrum disorder, cerebral folate deficiency, folate receptor alpha, folate receptor alpha autoantibodies, heritability

## Abstract

Autism Spectrum Disorder (ASD) affects an estimated prevalence of 1 in 31 children but the cause in most cases is unknown. Human and animal studies have linked ASD to Folate Receptor Alpha Autoantibodies (FRAAs). Our previous studies demonstrated that FRAAs are more common, on average, in families with children with ASD. This study reanalyzed data from a previous study which included 82 children diagnosed with ASD, 53 unaffected siblings, 70 mothers, 65 fathers, and 52 typically developing controls who did not have a history of ASD in their family. This study investigates the association of FRAA titers with ASD risk factors and explores the relationship of FRAA titers across generations. Several known risk factors for ASD, including multiplex ASD families, multiple birth pregnancies, and increased maternal and paternal ages at birth, were related to offspring FRAA titers. Multiplex ASD families demonstrated higher FRAA titers. Significant correlation were found between maternal and offspring blocking FRAA titers. FRAA titers increased across generations, although the increase in blocking FRAA titers was only seen in multiplex families. The proband with ASD showed higher blocking but not higher binding, FRAA titers compared to their non-affected siblings. Paternal FRAA titers are associated with several measures of offspring behavior and cognitive development. This research highlights the potential transgenerational transmission of FRAAs and their role in ASD. This supports the notion that heritable non-genetic factors may be important in the etiology of ASD and that FRAAs may demonstrate anticipation (worsening across generations), especially in multiplex families. FRAAs may provide one example of the possibility that susceptibility to autoimmune processes may contribute to disrupted brain development and function in ASD.

## 1. Introduction

Autism spectrum disorder (ASD) is a behaviorally defined neurodevelopmental disorder characterized by social-communication deficits with restrictive and repetitive behaviors and interests as outlined by the Diagnostic Statistical Manual of Mental Disorders Version 5 Text Revision [1]. The Centers for Disease Control and Prevention-funded Autism and Developmental Disabilities Monitoring Network has reported a continued increase in the prevalence of ASD over the past two decades with a current estimated prevalence of 1 in 31 children [2]. Despite decades of ASD surveillance and research, the cause of ASD remains uncertain in most cases [3].

ASD is highly heritable with studies finding a heritability of about 50% [4]. Having one child with ASD increases the chances of having another child with ASD. Indeed, there is an 80% recurrent risk for identical twins and 20% for nonidentical siblings [4]. Most commonly, heritable factors are thought to be transmitted genetically. However, inherited defects in single genes (e.g., SHANK3) are rare [5], with most single gene mutations being de novo, meaning that they are not inherited [6,7], and recent studies using whole genome sequencing report yields between 33% and 50% [8].

Non-genetic factors are also heritable. Specifically, many physiological abnormalities without a purely genetic etiology are found in both children with ASD and their mothers. These include mitochondrial [9,10,11], transmethylation/transsulfuration [12,13,14], and immune [15,16] abnormalities. This suggests that the heritable transmission of these abnormalities may not be purely genetic. These physiological systems are sensitive to environmental stressors, consistent with the notion that ASD may mainly be driven by genetic–environmental interactions [17] and linked to the maternal environment [18,19] and exposure to environmental factors [20].

**Figure 1:**
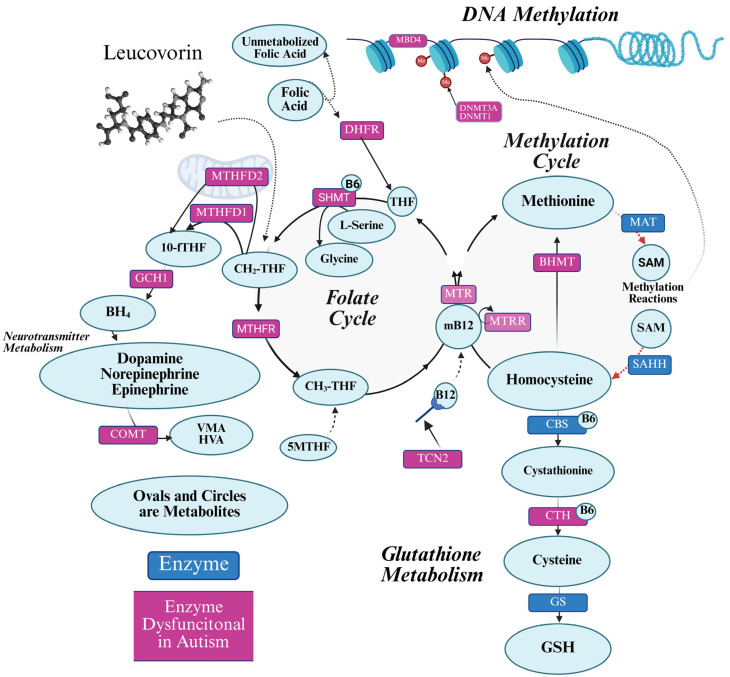
Cellular folate metabolism. The folate cycle is central to other critical cellular biochemical systems including methylation, glutathione production, and neurotransmitter production. The key enzymes for biochemical reactions are provided in the rectangles, with the enzymes having polymorphism overrepresented in ASD colored in purple. 5-MTHF/CH_3-_THF: 5-methyltetrahydrofolate; B12: Vit B12 (cobalamin); BH_4_: tetrahydrobiopterin; BHMT: Betaine-homocysteine methyltransferase; CBS: cystathionine beta-synthase; CH_2-_THF: 5,10-methylenetetrahydrofolate; COMT: Catechol-O-methyltransferase; CTH: cystathionine gamma-lyase; DHFR: dihydrofolate reductase; DNA: deoxyribonucleic acid; DNMT1: DNA methyltransferase 1; DNMT3A: DNA methyltransferase 3A; GCH1: GS: glutathione synthetase; GTP cyclohydrolase I; MAT: methionine adenosyltransferase; MBD4: Methyl-CpG-binding domain protein 4; Me: methyl group; MTR: Methionine synthase; MTHFD1: Methylenetetrahydrofolate dehydrogenase 1; MTHFD2: Methylenetetrahydrofolate dehydrogenase 2; MTHFR: methylenetetrahydrofolate reductase; MTRR: methionine synthase reductase; RNA: ribonucleic acid; SAHH: S-adenosylhomocysteine hydrolase; SHMT: Serine hydroxymethyltransferase; TCN2: Transcobalamin II; THF: tetrahydrofolate. Created in BioRender.

Folate is a water-soluble B vitamin (Vitamin B9) that is essential for normal neurodevelopment [21,22]. ASD is associated with abnormalities in methylation metabolism, including abnormal concentrations of key methylation metabolites such as methionine, S-adenosyl-L-methionine (SAM) and S-adenosyl-L-homocysteine (SAH) (see Figure 1). Deficits in SAM may be particularly important as SAM is the major methyl donor essential for deoxyribonucleic acid (DNA) and histone methylation, the epigenetic process that regulates gene expression. Consistent with this, postmortem brain studies show alterations in DNA methylation in the frontal cortex [23] and other brain regions [24,25] in individuals with ASD.

Defects in folate metabolism can cause the physiological abnormalities associated with ASD such as abnormalities in the purine, methylation, and redox metabolic pathways (Figure 1) [26]. Purines are essential for DNA synthesis, repair, and replication. Limitations in purine production can result in de novo mutations, chromosomal instability, copy number variation, and gross chromosomal abnormalities—all abnormalities associated with ASD [27,28,29]. Purine guanosine 5′-triphosphate (GTP) is the precursor of BH_4_ which is essential for monoamine neurotransmitter and nitric oxide production, both of which have been shown to be abnormal in ASD [30,31,32].

Several genetic folate pathway abnormalities associated with ASD are also found in mothers. For example, the same reduced folate carrier (RFC) single nucleotide polymorphism (SNP) that is overrepresented in children with ASD is also overrepresented in the mothers of children with ASD [33] and is linked to DNA hypomethylation in these mothers [33]. Furthermore, animal models have linked the maternal and fetal 677C > T methylenetetrahydrofolate reductase (MTHFR) SNP with abnormal development of inhibitory γ-Aminobutyric acid (GABA) pathways in offspring, resulting in ASD-like behavior [34].

**Figure 2:**
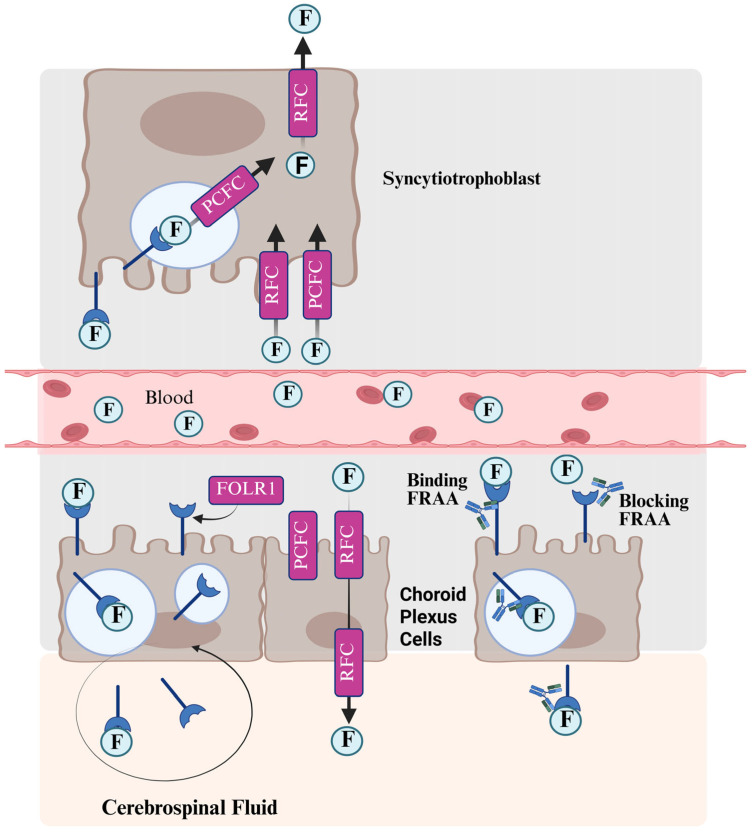
The transport of folate across the placenta and into the nervous system and the role of folate receptor alpha (FOLR1) autoantibodies (FRAAs) in disrupting this transport. F: folate, PCFC: protein coupled folate carrier, RFC: reduced folate carrier. Created in BioRender.

Although systemic folate abnormalities are associated with ASD, perhaps folate metabolism abnormalities have a more significant effect in ASD. In 2005, the senior author (EVQ) co-authored and published a pivotal study in the New England Journal of Medicine which demonstrated that some children with neurodevelopmental disorders suffered from a new disorder called cerebral folate deficiency (CFD), which was caused by autoantibodies to the folate receptor α (FRα), causing it to become nonfunctional [35] (Figure 2). Many of the patients in this study demonstrated symptoms of ASD. Subsequently, several case series linked CFD to low-functioning ASD with neurological deficits [36] and ASD with developmental regression, mental retardation, epilepsy, and dyskinesias [37]. In 2013, the first (REF) and senior (EVQ) authors found that 75% of children with ASD manifested one of the two FRα autoantibodies (FRAAs) [38]. A recent meta-analysis has confirmed this estimate [39].

More significantly, the first (REF) and senior (EVQ) authors published the first open-label [38] and double-blind placebo-controlled [38] studies in 2012 and 2016, respectively, demonstrating that the treatment for CFD, leucovorin (also known as folinic acid), resulted in significant improvements in children with ASD. Four placebo-controlled studies have confirmed these findings [40,41,42,43]. Most importantly, two of these placebo-controlled clinical trials demonstrated that FRAAs are predictive of responses to leucovorin [38,41]. It is believed that leucovorin uses the RFC, an alternative transporter, to enter the brain when the FRα is dysfunctional. The importance of FRAAs and leucovorin treatment in other developmental disorders in now being appreciated [44].

Quadros et al. [45] found that FRAAs were not only prevalent in individuals with ASD but also in other family members as compared to typically developing controls from families without children with ASD, a finding that verified other previous reports [39]. Interestingly, the nuances of the relationships to ASD and to potential transgenerational transmission remain understudied. In the current study, the data from Quadros et al. [45] is reanalyzed to determine whether FRAA titers are related to other ASD known risk factors such as being born in multiplex families, being born during a multiple birth pregnancy, or being related to maternal or paternal age.

Families with ASD are divided into two types, simplex and multiplex families. Simplex families have only one child with ASD, while multiplex families have more than one child with ASD. The fact that some families have many children with ASD suggests some type of family risk factor. Interestingly, although genetic disorders are highly heritable, simplex families have a higher rate of a genetic etiology for the ASD as compared to multiple families [46]. This suggests that multiplex families have a factor that increases the heritable risk of ASD that is not related to single genetic mutations.

A relationship between multiplex families and FRAAs could help examine the increased risk of ASD in these families, as genetic inheritance does not seem to examine this this phenomenon [46]. Indeed, when siblings have causative genetic mutations, they are only the same 31% of the time [47], suggesting that the primary etiology of genetic mutations is not the genetic mutation itself but a process driving the development of the genetic mutations, such as a folate pathway abnormality. Additionally, it is important to know whether FRAA titers increase across generations and whether parental FRAAs are related to the behavioral or cognitive outcome of the child with ASD.

We analyzed the data from our previous study to answer the following key questions:

• Whether FRAA titers are higher in multiplex vs. simplex families, as higher FRAA titers in multiplex families could explain the recurrent risk.
• Whether FRAAs are associated with increased parental age as such a relationship could explain the association of ASD with advanced parental age, especially advanced paternal age.
• Whether FRAAs are transmitted across generations and whether this transmission demonstrates anticipation (increase severity across generations), as anticipation could explain the increase in ASD prevalence in successive generations.
• Whether FRAAs in parents influence the cognitive and behavioral development of their offspring, as such a finding would highlight the importance of the role of parental FRAAs in neurodevelopment.

## 2. Results

### 2.1. Participant Characteristics

Table 1 provides the participants’ characteristics. Three children with ASD demonstrated gastrointestinal co-morbidities and nine had epilepsy. Eight children with ASD were being treated with neuroleptics, one was being treated with a stimulant, three were being treated with an antidepressant, three were being treated with an antiepileptic, and one was being treated with lithium. Most were receiving behavioral intervention, and none were being treated for folate metabolism abnormalities. There were 17 (22%) multiplex families and 8 (11%) with multiple births. Typically developing siblings include two half siblings, one male and one female.

### 2.2. Effect of Pregnancy and ASD Family Type on FRAA Titers

The effect of pregnancy factors, ASD family type (simplex vs. multiplex), and parental age on FRAA titers in offspring (ASD and siblings combined) was analyzed. The controls were not included in these analyses.

**Figure 3:**
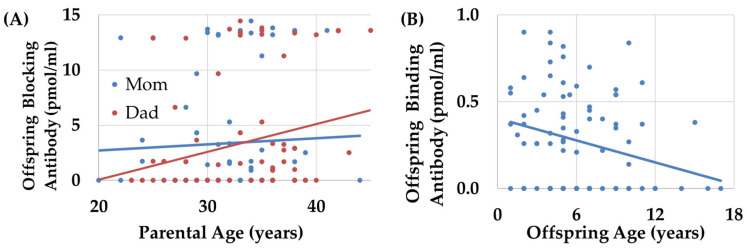
The relationship between age and FRAA titers. (**A**) Both maternal (blue line) and paternal (red line) ages were related to blocking FRAA titers such that higher levels of blocking FRAA titers in offspring were associated with a higher parental age. (**B**) Binding FRAA titers decreased with older offspring ages.

For probands and their siblings, multiplex families were found to have significantly higher blocking titers by 4.86 (1.13) pmol/mL as compared to simplex families with a large effect size [F(1,75) = 18.50, *p* < 0.001; Cohen’s *d′* = 1.1], with no significant effect found for sex, multiple birth pregnancies, or age at antibody measurement. Older maternal [F(1,75) = 9.18, *p* < 0.01; Cohen’s *d′* = 0.61] and parental ages [F(1,75) = 11.06, *p* = 0.001; Cohen’s *d′* = 0.67] at birth were related to higher blocking titers, both with medium effect sizes (see Figure 3A).

For probands and their siblings, multiple birth pregnancy was associated with a significantly higher binding titers as compared to single birth pregnancies with a medium-to-large effect size [F(1,109) = 3.88, *p* = 0.05, Cohen’s *d′* = 0.66] and binding titers decreased with the age at which the binding antibody was measured, with a medium effect size [F(1,109) = 8.546, *p* < 0.01; Cohen’s *d′* = 0.58] and with no significant effect found for sex or multiplex families (Figure 3B).

### 2.3. Transgenerational Changes in FRAA Titers

**Figure 4:**
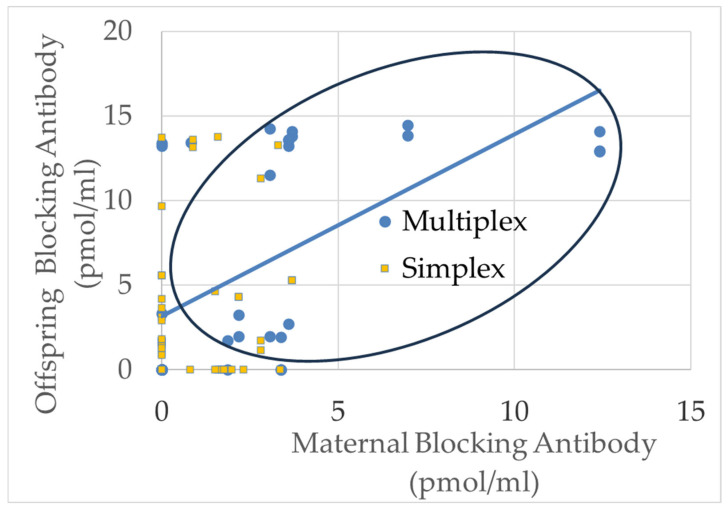
The relationship between offspring and maternal blocking FRAA titers. The blue circles represent multiplex families while the orange squares represent simplex families. Oral highlights the cases in which both offspring and maternal blocking FRAA titers were positive. These cases are disproportionally from multiplex families.

Offspring (ASD and Sibling) blocking titers were significantly related to maternal blocking titers [F(1,105) = 33.514, *p* < 0.001, Cohen’s *d′* = 1.16] (Figure 4), but binding offspring FRAA titers were not found to be related to parental FRAA titers.

The relationship between blocking FRAAs in offspring and their mothers was clearly driven by a group in which titers were high for both the offspring and the mother (point in the oval in Figure 4) and another group where the blocking FRAA was only elevated in the offspring OR the mother. As the mean titers were higher for the multiplex families, it was hypothesized that these double positives (offspring, mother) were driven by multiplex families. For the blocking FRAAs, 53% of the multiplex families demonstrated positivity in both offspring and mother, whereas this was only seen in 12% of the simplex families. This difference was highly significant, with a large effect size [χ^2^ = 24.47, *p* < 0.0001, Cohen’s *d′* = 1.14].

**Figure 5:**
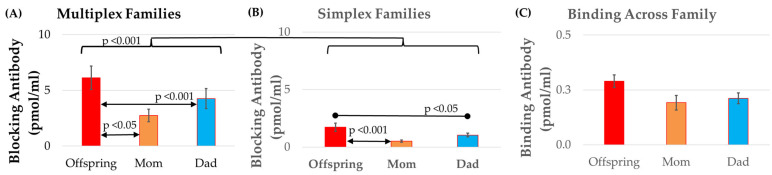
Relationship of FRAA titers between parents and offspring. The change in blocking titers across generations was significantly higher in (**A**) multiplex as compared to (**B**) simplex families. For both multiplex and simplex families, offspring blocking titers were significantly higher than parental blocking titers. (**B**) The titers for the offspring, mothers, and fathers were similar in simplex families. (**C**) Binding titers were not significantly difference across family members.

Comparing offspring to parental blocking titers, the multiplex families (Figure 5A) manifested significantly higher titers than the simplex families (Figure 5B) by 3.73 (0.73) pmol/mL, with a large effect size [F(1,72) = 26.50, *p* < 0.001, Cohen’s *d′* = 0.82], and the offspring blocking titers were significantly greater than the maternal [F(1,200) = 5.91, *p* < 0.05, Cohen’s *d′* = 0.39] and paternal [F(1,200) = 6.74, *p* < 0.001, Cohen’s *d′* = 0.65] blocking titers by 1.06 (0.44) and 1.78 (0.44), with a small and medium effect size, respectively. Binding titers were not significantly different across family members or between simplex and multiplex families (see Figure 5C).

### 2.4. Effect of ASD on Family Titers

To determine the effect of ASD over and above the effect of being an offspring, a mixed-model with separate factors for offspring and children with ASD was used to examine FRAA titers across generations.

Blocking titers were significantly higher by 1.62 (0.50) [F(1,215) = 10.60, *p* < 0.001, Cohen *d′* = 0.46] in offspring as compared to parents and being affected by ASD increased the titer by 3.92 (1.08) [F(1,215) = 5.92, *p* < 0.05, Cohen *d′* = 0.40], with both having a small-to-medium effect size. Multiplex families demonstrated blocker titers higher by 3.44 (0.75) [F(1,74) = 21.09, *p* < 0.001, Cohen’s *d′* = 0.74], with a medium-to-large effect size. Multiple birth pregnancy interacted with the affected individual such that a child with ASD demonstrated a significantly higher titer if they were born from a multiple birth pregnancy [F(1,211) = 16.95, *p* < 0.001, Cohen’s *d′* = 0.67], with a medium effect size.

Binding titers were significantly higher by 0.08 (0.04) [F(1,208) = 4.24, *p* < 0.05, Cohen’s *d′* = 0.34] in offspring as compared to parents, with a small effect size, but being affected by ASD did not significantly change FRAA titer.

### 2.5. Familial FRAA Titers Compared to Controls

**Figure 6:**
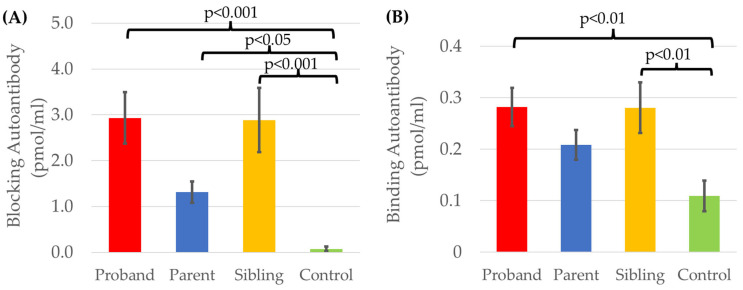
Comparison of (**A**) blocking and (**B**) binding FRAA titers in proband families and typically developing controls.

Participant groups (proband, sibling, parent, controls) differed in terms of blocking titers [F(3,272) = 10.51, *p* < 0.001, Cohen’s *d′* = 0.38] with a small-to-medium effect size, and binding titers [F(3,278) = 3.93, *p* < 0.01, Cohen’s *d′* = 0.23] with a small effect size (Figure 6). Post hoc comparisons using two-sided Dunnett t found that blocking titers were significantly higher in the families of children with ASD as compared to the typically developing controls. Binding titers were significantly higher in the children with ASD and their siblings as compared to the typically developing controls who did not have a sibling with ASD.

### 2.6. Behavior and Development

The participants with ASD were assessed using several instruments to determine their development and ASD symptoms. A mixed model controlling for family unit was used to determine whether the presence of FRAAs (positive vs. negative) in the moms or dads was associated with development and/or ASD symptoms. The presence of blocking and binding FRAAs in a child was included in the model to control for any effect of FRAAs on the child. The results are summarized in Table 2.

A positive FRAA status for the father was related to poorer development and greater ASD symptoms in the child, with medium to large effect sizes, as described below. Several of the Griffith’s Mental Development Scales, which were performed on the subset of children within the range of the test (≤8 years of age), were lower when the father was FRAA positive. This included the total developmental score (Cohen’s *d′* = 0.66), motor development (Cohen’s *d′* = 0.69), speech and language development (Cohen’s *d′* = 0.85), coordination (Cohen’s *d′* = 0.66), and practical reasoning (Cohen’s *d′* = 0.64), all with medium effect sizes. The VABS demonstrated poorer development in communication (Cohen’s *d′* = 0.47), socialization (Cohen’s *d′* = 0.73), daily living skills (Cohen’s *d′* = 0.73), and motor skills (Cohen’s *d′* = 0.48) if the father was FRAA positive, with medium-to-large effect sizes.

ASD symptoms were assessed using the PDDBI, which demonstrated more severe ASD symptoms if the father was FRAA positive; specifically, a positive FRAA status in the father was related to a worse overall PDDBI score (Cohen’s *d′* = 0.46), as well as worse Expressive Language (Cohen’s *d′* = 0.46), Learning, Memory, and Receptive Language (Cohen’s *d′* = 0.45), Expressive Social Communication Abilities Composite (Cohen’s *d′* = 0.40) and Receptive/Expressive Social Communication Composite scores (Cohen’s *d′* = 0.46). A Positive FRAA status in the mother was associated with worse Social Pragmatic Problems (Cohen *d′* = 0.61), all with medium effect sizes.

## 3. Discussion

This paper examines the transgenerational heritability of FRAAs particularly with respect to autism spectrum disorder (ASD), one of the major disorders which with it has become associated. The many interesting aspects of this study include the fact that transgenerational heritability appears to be slightly different for the blocking and binding FRAAs. Of note, two previously unrecognized factors that affect the FRAA titers of offspring, multiplex ASD and multiple births, were found. Interestingly, the FRAA status of the father appears to have a relationship with the outcome of a child with ASD. The findings from this study are summarized in Table 3.

Although multiple birth and multiplex families represented a minority of the sample, it was found that these families demonstrated a distinct relationship with FRAA titers. Both factors are risk factors for ASD.

Families with multiple birth pregnancies manifested higher FRAA binding titers. Specifically, a multiple birth pregnancy is a risk factor for the offspring developing ASD [48], possibly because of perinatal and neonatal complications which are also associated with ASD [49,50].

Multiplex families were found to have an overall higher blocking titer as compared to simplex families, and the risk of having another child with ASD is higher in multiplex families [51], suggesting that FRAAs may be involved in the biological mechanisms which increase ASD risk, especially since multiplex families are less likely to have an identifiable genetic cause for ASD, suggesting other non-genetic factors, such as FRAAs, may be playing a role in these families [46]. The affected children with ASD in the multiplex families demonstrated significantly higher titers than the other typically developing family members, thus making it a factor which distinguishes a child with ASD from their typically developing family members. Interestingly, the fact that multiplex families have higher FRAA titers than simplex families is consistent with several studies which demonstrate that the members of multiplex families have subtle cognitive deficits. The typically developing siblings from multiplex families, where FRAA titers were higher, tend to have lower cognitive function and adaptive behavior as compared to typically developing siblings from the simplex families [52]. Family members in multiplex families exhibited more ASD characteristics than family members in simplex families [53,54,55,56]. Although the number of multiplex families was lower than the number of simplex families, the proportion of the samples of multiplex families was similar to other studies comparing multiplex and simplex families [52]. Nevertheless, these are compelling findings need to be confirmed in larger samples.

Another compelling finding is that FRAA blocking titers increased with parental age, particularly that of the father. Such a finding is consistent with the association of advanced parental age with risk factors for ASD. The association between advanced maternal age and several reproductive issues, such as infertility and fetal malformations, is well known. FRAAs have been proposed to be associated with pregnancy complications given the high concentration of folate receptors at the maternal–fetal interface [57] and the association between FRAAs and neural-tube defects [58,59,60], preterm birth [61], and subfertility [62]. The association of ASD with advanced paternal age is somewhat unique for ASD. Studies suggest that this is due to decreased sperm quality and testicular function. Although the FRα is not known to be located in testes, one major role of the epididymis and vas deferens is to secrete a folate-binding protein which adheres to spermatozoa [63]. The potential cross reaction between FRAAs and the folate-binding protein could affect sperm quality.

Blocking but not binding titers were correlated between mother and offspring. A closer examination demonstrated that the effect was driven by cases in which both mother and offspring had high blocking FRAA titers. These cases were overrepresented in multiplex families, suggesting that multiplex families are more likely to have maternal–offspring heritability of FRAAs, whereas the simplex families tend to have positive blocking FRAAs in either the mother OR offspring but not both.

Interestingly, both binding and blocking titers increased across generations. This phenomenon of increasing severity of a disease across generations has been termed anticipation in genetics where it is used to describe an increase in the severity of trinucleotide repeat disorders across generations. Like genetic anticipation, in which a gene abnormality can become worse until it reaches a threshold to cause disease, FRAAs appear to increase in severity (titer concentration) across generations. It may very well be that the FRAA titer is required to be at a specific level to cause disease, so ASD may not be manifested until the titer reaches a certain critical level. However, it is more likely that ASD symptoms are less severe in family members with FRAAs such that they do not severely interfere with life. The mechanism by which FRAA titers increase across generations is not known and will require further research.

We found several developmental characteristics related to paternal FRAA status, including measures of mental development, ASD behavior, and adaptive functioning. As shown in Table 2, the PDDBI data largely replicated the Griffiths and Vineland results and indicated that the primary effect was on the domains and composite scores assessing social communication competence (a core feature of ASD), rather than on the domains and composite scores assessing pragmatic problems, repetitive/ritualistic behaviors, and arousal/anxiety problems. In addition, the PDDBI also emphasizes the language defect associated with FRAA status.

As previously discussed, it is not clear how a paternal FRAA status affects the development of offspring, but there are several potential mechanisms. First, the FRα is secreted into the semen where it interacts with spermatozoa [64]. A glycolipid linked high-affinity folate-binding protein is secreted from the epithelium of the epididymal and vas deferens where it associates with prostasome-like vesicles adherent to spermatozoa [63]. This folate-binding protein is believed to have a bacteriostatic function or facilitates folate transport into the spermatozoa [63]. FRAAs could bind to this folate-binding protein, possibly interfering with the development of high-quality sperm. Second, FRAAs in the semen could also bind to JUNO (a pseudo-folate receptor known as FOLR4) on the oocyte during fertilization [65].

While folate metabolism abnormalities could have physiological effects on biomedical pathways (as discussed in the introduction) perhaps most importantly, folate disruption can result in genetic [66] and epigenetic [67] alternations that have been linked to ASD [68]. ASD is associated with single nucleotide de novo variants, which are most likely to occur during spermatogenesis, which commences in adolescence and continues throughout life. Toxicant exposure and poor folate intake throughout life could certainly result in a cumulative mutation load, resulting in poorer sperm quality with age. This not only implies the importance of paternal folate intake but also provides an explanation of the relationship between advanced paternal age and an increased risk of ASD. This study found an increase in FRAA titers in the offspring with paternal age, suggesting a proper intake of reduced folates is important, especially in males as they age.

Disruptions in folate metabolism have been linked to epigenetic changes, particularly DNA demethylation, in genes associated with brain development [67], resulting in abnormal gene expression [69]. Furthermore, environmental exposures such as air pullulation as well as lifestyle factors provoke changes in DNA methylation and gene silencing [70]. Evidence is building that these changes are transgenerational, with environmental and lifestyle factors in parents affecting the development of their offspring [71].

It is known that folate is protective against the detrimental effects of environmental factors on pregnancy, including air pollution [72] and heavy metals [73], reduces pregnancy complications including preterm birth, pre-eclampsia, placental abruption, fetal growth restriction, and fetal death [74] and reduces the risk of ASD [75]. Folate supplementation during pregnancy reduced the environmental exposure-related ASD risk by half [76], demonstrating its importance in supporting detoxification pathways during pregnancy. Folate’s protective effect against ASD during pregnancy seems to be greatest in early pregnancy [75,77], when neurogenesis is rapid. Low maternal folate levels during pregnancy increase the risk of cognitive and behavioral neurodevelopmental disorders [78] and language developmental disorders [79].

The significance of prenatal FRAAs being associated with ASD behavior identified in this study confirms the findings of previous animal models. Indeed, in previous animal studies, exposure of the dam to FRAAs resulted in an accumulation of FRAAs in the placenta and the fetus, and blocked folate transport to the fetal brain, resulting in severe behavioral deficits in the offspring [80]. Further studies demonstrated that these behavioral defects could be prevented by treatment with high doses of leucovorin (folinic acid) during gestation [81]. Additionally, other animal studies found that high doses of folate could restore folate delivery to the placenta and fetus in the context of FRAAs, with leucovorin and levoleucovorin demonstrating the best distribution [82]. In a clinical series, parental FRAAs were found to be associated with more severe ASD [83]. FRAAs during pregnancy in humans have been associated with neural-tube defects [84] and miscarriages [57].

Collectively, this study and the supporting literature suggests that high dose folate supplementation during and before pregnancy may be therapeutic in future parents with FRAAs. Animal studies have demonstrated that leucovorin/levoleucovorin appears to be optimal for the delivery of folate to a fetus in the context of FRAAs [82]. It was noted that because levoleucovorin generated higher tetrahydrofolate levels than other folates and can readily incorporate into the folate cycle without the need for MTHFR or methionine synthase in the first pass, it should be the folate of choice for the treatment of individuals with FRAAs [82]. Additionally, leucovorin is transformed into 5-MTHF in the gastrointestinal tract [85]; so, when supplied orally, the body receives THF, 5-MTHF, and 5-formyl tetrahydrofolate, making any MTHFR polymorphism irrelevant.

Lastly, it is important to note that in the context of folate metabolism abnormalities, adequate levels of folate cannot be achieved by folic acid supplementation. Folic acid is a synthetic form of folate that is not found in nature and cannot be used by metabolism until it is converted into a reduced form. The gastrointestinal tract has a limited capacity to reduce folic acid [85] as does the liver [86]. High concentrations of folic acid will saturate the DHFR enzymes leading to unmetabolized folic acid (UMFA). It is estimated that oral doses of folic acid in excess of about 280 μg lead to UMFA in the systemic circulation [87]. Studies have suggested that UMFA can cause health risks such as immune dysfunction [88,89] and UMFA in maternal blood has been linked to ASD [77]. Thus, it is important to avoid supplementation with high amounts of folic acid.

This study provides preliminary evidence for family factors that might be related to FRAAs. However, other factors that have not been measured might affect FRAA titers. For example, FRAA titers are reduced by a milk free diet and it is possible that extrinsic folate, provided by fortification or supplementation, could influence the blood concentrations of FRAAs. Additionally, children with ASD have an overexpression of SNPs in one-carbon metabolism pathway genes. Such SNPs could modulate folate metabolism and FRAA concentrations. Future research with larger cohorts, a balanced number of simplex and multiplex families, and sex- and age-matched controls without ASD could also improve the study design and validate these findings.

## 4. Materials and Methods

Data were derived from our previous published study on the family prevalence of FRAAs [45]. The participants with ASD were recruited as part of a study of ASD at the Institute for Basic Research in Developmental Disabilities (IBR), Staten Island, New York between the years 2000 and 2017. Diagnosis of ASD was established based on DSM-IV [90]; DSM-IV-TR [91] and DSM-5 [92] criteria using information from the Autism Diagnostic Interview—Revised and Autism Diagnostic Observation Scale, the two gold standards for ASD diagnosis, and parental interviews. Unrelated, controls without ASD were recruited from a local pediatric practice.

The protocol was approved by the Institutional Review Board at IBR (Staten Island, NY, USA). The parents of participants provided written informed consent. Data were then deidentified for analysis. All dates were transformed to age in days at the time of visit and personal health identifiers were removed to deidentify the data for further analysis. Thus, the final dataset was analyzed under 45 CFR 46 exemption.

### 4.1. Titer Measurement

The FRAA assay was performed by the laboratory of Dr Quadros at SUNY Downstate. An in vitro functional blocking assay was used to measure blocking FRAAs, while an enzyme-linked immunosorbent assay (ELISA) specific for binding IgG was used to measure binding FRAAs, as previously described [93].

### 4.2. Developmental Assessments

The Griffiths Scales of Child Development (1984) provide an overall measure of development for children from infancy to 8 years of age [94]. It includes subscales for assessing learning, language, and communication; eye and hand coordination; personal, social, and emotional function; and gross motor function. It is used by a trained examiner interacting with a child. Age standardized scores (MA/CAX 100) are provided with lower scores representing worse development.

The PDD Behavior Inventory (PDDBI; 2005) is a reliable and valid caregiver questionnaire assessment tool which measures both problem behaviors and social communication abilities [95]. It provides age-standardized T-scores based on a large sample of well-diagnosed children with ASD. It is divided into two dimensions: Approach/Withdrawal problems and Receptive Expressive Social Communication Abilities along with an overall Autism Composite score. For the Approach/Withdrawal problems dimension and the Autism Composite dimension, higher scores indicate greater severity. For the Receptive Expressive Social Communication Abilities dimension, higher scores indicate greater competence.

The Vineland Adaptive Behavior Social Subscale III (VABS) is a widely used standardized, well-validated assessment tool for children with developmental delays that measures functional abilities. It is a valid measure of the social impairments in children with ASD [96]. The VABS relies on an informant (caretaker) to complete. Higher scores represent better development.

Gross motor development was assessed with the PDDBI in a subset of parents by asking the parents to note at which age their child sat and walked without support, as these are milestones that are typically remembered well by parents.

### 4.3. Statistical Analysis

Analyses were performed using PASW Statistics version 28.0.0.0 (IBM SPSS Statistics, Armonk, NY, USA). Graphs were produced using Excel version 14.0 (Microsoft Corp., Redmond, WA, USA). An alpha of 5% was used as a cutoff for significance. Means are presented with standard deviations followed in brackets.

In general, mixed-model regression models with the random effects of family (the shared variance among parents and sibling) were used to account for the family level mean and variance. Analysis included dichotomous variables such as a multiplex family, multiple births, and sex and continuous variables such as offspring, as well as maternal and paternal ages. Two-way Interactions between variables were included in the model. The final model was simplified to only significant variables and variables that were dependent on significant interactions. Cohen’s *d′* was calculated to represent effect size. Effects from model coefficients are provided for dicrotous variables with standard errors.

## 5. Conclusions

This study examined the relationship between FRAAs in families to help better define their role in reproductive health and the neurodevelopmental outcomes of offspring. Several novel findings were found, including a relationship between FRAA titers and both multiplex families and multiple birth pregnancies, two factors that are known to increase the risk of ASD in offspring. The blocking FRAA appears to be highly correlated between mothers and their offspring, thereby supporting the notion of a relationship across generations. Finally, the notion of the anticipation of FRAAs across generations is compelling and may suggest an increased risk of ASD in parents that have higher titers. This study is limited, mostly by its sample size. Larger studies will be needed to verify and further this research.

## 6. Patents

EVQ and JMS are inventors on a US patent for the detection of FRAAs issued to the Research Foundation of the State University of New York, NY, USA.

## Figures and Tables

**Table 1:** Participant characteristics.

	N	Sex [% Male (N)]	Age Range (Years)	Average Age (SD)
ASD	82	79% (65)	1.6 to 15	5.18 (2.6)
Typically Developing Siblings	53	42% (22)	2 to 17	6.7 (4.2)
Parents of Children with ASD	135	48% (65)	15 to 54	32.4 (5.8)
Unrelated, non-ASD controls	52	46% (24)	3 to 17	11.8 (4.0)

**Table 2:** Relationship between FRAA status in moms and dads and the cognitive development and behavior of their offspring with ASD. Means and standard errors of the offspring’s cognitive and behavioral symptoms are given for the positive and negative FRAA statuses of the parents. Statistically significant differences are emboldened. The number of participants for each evaluation are given in the instrument name header. The subscales of the main composite scale are indented under the main composite scale title. * *p* ≤ 0.05; ** *p* ≤ 0.01.

	Mom		Dad	
Griffth’s Mental Development Scales				
	Neg N = 18	Pos N = 29	Neg N = 10	Pos N = 32
Total Scale (GQ)	69.1 (5.2)	75.4 (3.9)	85.2 (8.5) *	**70.4 (3.4)**
Locomotor Development	83.9 (3.6)	86.6 (2.5)	93.5 (6.8) *	**83.4 (1.9)**
Personal Social Development	64.6 (5.2)	65.3 (3.9)	74.0 (8.3)	63.0 (3.2)
Hearing and Speech	54.8 (6.9)	61.3 (6.9)	80.8 (15.0) **	**51.9 (27.7)**
Hand and Eye Coordination	68.3 (5.7)	76.9 (4.1)	85.1 (7.8) *	**71.2 (3.8)**
Performance	80.9 (6.4)	88.2 (4.0)	96.5 (7.4)	83.9 (4.2)
Practical Reasoning	63.2 (6.3)	69.0 (5.2)	81.2 (11.0) *	**63.3 (4.2)**
PDD (Pervasive Developmental Disorder) Behavior Inventory				
	Neg N = 41	Pos N = 63	Neg N = 25	**Pos** N = 72
Autism Composite	49.1 (1.7)	51.0 (1.3)	45.9 (1.8) *	**51.3 (1.2)**
Sensory/Perceptual Approach Behaviors	51.4 (1.5)	48.7 (1.2)	46.7 (1.7)	50.4 (1.2)
Ritualisms/Resistance to Change	48.3 (1.3)	50.3 (1.3)	45.9 (1.6)	49.6 (1.2)
Social Pragmatic Problems	47.0 (1.6) **	**53.1 (1.1)**	48.9 (1.7)	51.1 (1.2)
Semantic/Pragmatic Problems	51.5 (2.1)	48.8 (1.1)	49.7 (2.2)	49.2 (1.1)
Arousal Regulation Problems	52.3 (1.5)	50.3 (1.3)	49.9 (1.8)	51.1 (1.2)
Specific Fears	48.8 (1.5)	50.7 (1.2)	47.7 (2.1)	50.7 (1.1)
Aggressiveness	48.8 (1.3)	50.9 (1.6)	48.5 (2.5)	49.5 (1.1)
Repetitive, Ritualistic, and Pragmatic Prob Comp	50.7 (1.8)	50.5 (1.2)	48.4 (1.9)	50.5 (1.2)
Approach/Withdrawal Problems Composite	50.6 (1.9)	51.3 (1.3)	49.4 (2.3)	50.7 (1.3)
Social Approach Behaviors	50.0 (1.8)	48.4 (1.2)	51.7 (2.0)	47.3 (1.2)
Expressive Language	50.1 (1.6)	49.1 (1.2)	53.4 (1.9) *	**47.8 (1.1)**
Learning, Memory, and Receptive Language	50.1 (1.6)	49.6 (1.1)	53.0 (2.0) *	**48.2 (1.0)**
Expressive Social Comm Abilities Composite	50.0 (1.8)	49.6 (1.2)	52.7 (1.9) *	**48.0 (1.2)**
Receptive/Expressive Social Comm Composite	50.0 (1.8)	49.7 (1.2)	52.7 (1.9) *	**48.0 (1.2)**
Vineland Adaptive Behavior Scales				
	Neg N = 29	Pos N = 44	Neg N = 19	Pos N = 49
Communication	71.0 (4.7)	63.7 (3.3)	77.5 (5.8) *	**63.4 (3.3)**
Daily Living Skills	62.0 (3.2)	58.0 (2.2)	69.5 (4.7) **	**56.5 (1.7)**
Socialization	66.1 (3.3)	60.3 (1.9)	72.1 (4.5) **	**59.3 (1.8)**
Motor Skills	70.2 (4.0)	70.8 (2.4)	79.6 (5.2) *	**67.7 (2.4)**
Developmental Gross Motor Milestones				
	Neg N = 15	Pos N = 21	Neg N = 10	Pos N = 26
Age when sat without support	6.7 (0.3)	6.9 (0.3)	6.5 (0.2)	7.1 (0.3)
Age when walking without support	13.4 (3.1)	14.0 (0.7)	12.8 (0.7)	14.4 (0.7)

**Table 3:** Summary of findings. Offspring includes children with ASD and their siblings. ASD families refer to families with at least one child with ASD.

Factor	Impact on Folate Receptor Alpha Autoantibodies
Multiple Birth Pregnancy	Higher Binding in the Offspring
Multiplex ASD Families	Higher Blocking in the Offspring, Mother, and Father
Offspring Age	Lower Binding in the Offspring
Offspring	Higher Blocking and Binding than Parents Higher Binding than Typically Development Children from Non-ASD Families
ASD	Higher Blocking than Parents and Typically Developing Sibling
ASD Families	Higher Blocking than Typically Development Children from Non-ASD Families
Increased Maternal Age	Higher Blocking in the Offspring
Higher Maternal Blocking	Higher Offspring Blocking
Maternal FRAA Positive	Greater Pragmatic Problems
Increased Paternal Age	Higher Blocking in the Offspring
Paternal FRAA Positive	Lower general intelligence, locomotor activity, hearing and speech, hand and eye coordination, practical reasoning skills, learning, memory, receptive and expressive language and social communication, and daily living, socialization, and motor skills. Greater ASD symptoms

## Data Availability

Data are available upon request.

## Abbreviations

The following abbreviations are used in this manuscript:

5-MTHF/CH_3-_THF	5-methyltetrahydrofolate
ASD	Autism Spectrum Disorder
B12	Vit B12 (cobalamin)
BH_4_	Tetrahydrobiopterin
BHMT	Betaine-homocysteine methyltransferase
CBS	Cystathionine beta-synthase
CFD	Cerebral folate deficiency
CH_2-_THF	5,10-methylenetetrahydrofolate
COMT	Catechol-O-methyltransferase
CTH	Cystathionine gamma-lyase
DHFR	Dihydrofolate reductase
DNA	Deoxyribonucleic acid
DNMT1	DNA methyltransferase 1
DNMT3A	DNA methyltransferase 3A
ELISA	Enzyme-linked immunosorbent assay
F	Folate
FOLR1	Folate receptor alpha
FRAAs	Folate Receptor Alpha Autoantibodies
FRα	Folate Receptor Alpha
GABA	γ-Aminobutyric acid (GABA)
GCH1	GTP cyclohydrolase I
GS	Glutathione synthetase
GTP	Guanosine 5′-triphosphate
IBR	Institute for Basic Research in Developmental Disabilities
MAT	Methionine adenosyltransferase
MBD4	Methyl-CpG-binding domain protein 4
Me	Methyl group
MTHFD1	Methylenetetrahydrofolate dehydrogenase 1
MTHFD2	Methylenetetrahydrofolate dehydrogenase 2
MTHFR	Methylenetetrahydrofolate reductase
MTR	Methionine synthase
MTRR	Methionine synthase reductase
PCFC	Protein-coupled folate carrier
PDDBI	Pervasive Developmental Disorder Behavior Inventory
RFC	Reduced folate carrier
RNA	Ribonucleic acid
SAH	S-adenosyl-L-homocysteine
SAHH	S-adenosylhomocysteine hydrolase
SAM	S-adenosyl-L-methionine
SHMT	Serine hydroxymethyltransferase
SNP	Single nucleotide polymorphism
TCN2	Transcobalamin II
THF	Tetrahydrofolate
VABSs	Vineland Adaptive Behavior Scales
SD	Standard Deviation
USA	United States of America
SUNY	State University of New York
